# Study of Soil Seed Banks in Ex-closures for Restoration of Degraded Lands in the Central Rift Valley of Ethiopia

**DOI:** 10.1038/s41598-020-57651-1

**Published:** 2020-01-22

**Authors:** Shemsedin Ahmed Mohammed, Mekuria Argaw Denboba

**Affiliations:** 1 Department of Environmental Science, College of Natural and Computational Science, Wolaita Sodo University, Wolaita Sodo, Ethiopia; 2 Center for Environmental Science, College of Natural and Computational Science, Addis Ababa University, Addis Ababa, Ethiopia

**Keywords:** Forest ecology, Ecosystem services

## Abstract

Soil seed banks (SSB) can be important components in the process of rehabilitating degraded lands. Thus, this study was aimed to evaluate the role of SSB to the restoration of degraded lands in six, fifteen and twenty-five year’s ex-closures and adjacent degraded open grazing land (DOGL). Totally, 160 samples of SSB from four soil layers (litter, 0–3 cm, 3–6 cm & 6–9 cm), four sites and ten in a composite of 5 (15 × 15 cm) were collected and tested for seed viability through seed extraction and seedling emergence methods. Species richness and diversity showed a significant difference between the SSB of the ex-closures and DOGL (P = 0.0148 and P = 0.0218 respectively). Seed densities also showed significant differences between the vertical layers of the soils in the ex-closures and DOGL (P = 0.0112) and the interaction effect of the land use type and the vertical soil layers (P = 0.0174). Ex-closures and DOGL scored highest seed densities in their litter and bottom layers of the soils respectively. Most of the woody species in the SSB of older ex-closures were represented in the aboveground flora. Thus, this study has verified that SSB has played a vital role in the restoration of woody species in degraded land through ex-closure practices.

## Introduction

Large areas of the world’s land have been degraded and landscapes everywhere are being simplified by different land-use practices^1^ and it became one of the most challenging concerns of environmentalists. Land degradation in Ethiopia through deforestation, agricultural land expansion, overgrazing, cutting of trees for fuelwood and construction materials is one of the national and local environmental challenges^2–4^. Avoiding the stress and allowing the degraded land to recover readily by itself had perceived as the most important tool next to the preservation of the original habitats and it has been widely recognized as a means of preventing the continual loss of biodiversity and receiving considerable attention. Hence, counteracting the degradation of vegetation to rehabilitate degraded lands had been one of the central concerns of the government and non-government organizations in Ethiopia^5^.

Therefore, government and non-government organizations have been promoted and started rehabilitation practices on the degraded lands of the country using a set of passive rehabilitation measures particularly ex-closures were dominantly practiced^2,4,5^. Thus, ex-closures are rehabilitation techniques where, degraded land areas are closed off from the interference of human and domestic animals to lessen land degradation of formerly degraded open grazing lands, promoting natural regeneration of plants and replenish biodiversity and restore soil fertility^5–7^. In ex-closures practices, it is believed that a degraded ecosystem recovers readily by itself in the absence of stress or disturbance through what is termed as “passive restoration”^8^.

In the passive restoration of degraded lands, the presence of viable soil seed banks and changes in environmental conditions such as moisture, light regime, and temperature fluctuations are very essential which may bring about the germination of dormant seeds that have remained viable in the soil for several years^9^. This implied that species overcome periods of unfavourable weather conditions by building up large seed stock in the soil, which is known as “soil seed banks”. This strategy protects plant species diversity against local extinction of the species during the disturbance and provides information on the past population dynamics and structure and future regeneration potential of degraded land^10^. Thus the “soil seed banks” are defined as an aggregation of viable seeds in the soil and the upper litter of the soil which are capable of replacing adult plants in a degraded ecosystem and a key factor for ecosystem reclamation^11–14^. Therefore, for a good result to be noted in the restoration process, gathering information on the presence and absence of persistent soil seed banks or sources of re-growth after disturbances are very crucial^15^.

Nowadays, area ex-closure is one of the most widespread forms of restoration practices in Ethiopia and as many studies in the northern part of Ethiopia have confirmed that the biophysical conditions of degraded open grazing lands had been recovered and made socioeconomic benefits to the local communities^4,16–19^. However, studies on the contribution of soil seed banks to the rehabilitation of degraded lands, particularly in area ex-closure practices were lacking. Thus, there were no documented studies on the status of soil seed banks in the degraded lands before the establishment of ex-closures and after the establishment of ex-closures thereby its role in the restoration process. However, research-based knowledge on such matters is very essential in planning the restoration of degraded land through ex-closure practices, and this study was designed to reduce the knowledge gap on the status of woody species in the soil seed banks of degraded open grazing land and ex-closures of different ages so the role of soil seed banks to a restoration process. Therefore, in Alaba district in the central rift valley of southern Ethiopia, ex-closures with six years, fifteen years and twenty-five years duration from their establishments in Choroko-1, Hebibo and Asore peasant associations respectively were selected for this study. Besides, adjacent to the ex-closures degraded open grazing land (DOGL) in Keranso was selected. Thus, the degradation status of DOGL in Keranso was with the assumption of in a similar status of degradation with the above-mentioned ex-closures before the establishment of the ex-closures.

Thus, the general objective of this study was to investigate the role of soil seed banks in the restoration of degraded lands through ex-closure practices in the central rift valley, southern Ethiopia. The emphasis was to: (1) Determine the composition, density, diversity and richness of woody species in the soil seed banks of six, fifteen, twenty-five years ex-closures and DOGL, (2) Investigate the soil seed bank distribution of woody species in the vertical layers of the soils in the six, fifteen, twenty-five years ex-closures and DOGL, (3) Examine and compare the similarities between woody species richness and diversity in the soil seed banks and their respective aboveground standing woody species in the six, fifteen, twenty-five years ex-closures and DOGL thereby to determine the role of soil seed banks to the restoration of woody species in the ex-closures.

## Results

### Species composition of soil seed banks

A total of 16 woody species in 12 families were recovered from the litter and the top nine centimetres of the soil samples collected from all the ex-closures and DOGL (Supplementary Table S1). From this total eight, seven, twelve and ten species were recovered from DOGL, six, fifteen and twenty-five years ex-closures respectively. While, six, seventeen, twenty-four and thirty-three aboveground standing woody species were recorded from the aboveground sample vegetation inventory of the DOGL, six, fifteen and twenty-five years ex-closures respectively. Fabaceae was represented by four species (25%) of all woody species in the soil seed banks of ex-closure and DOGL. The remaining 11 families were represented by only one species each. The higher proportion (75%) of the total species were trees and shrubs constitute only 25% of the life forms of the woody species in the soil seed banks. *Acacia torotolis* (Forssk.) Hayne*, Calpurnia aurea* (Ait.) Benth and *Dodonaea angustifolia* L.f were the only species detected in all the sampling sites.

Planted woody species during the establishment of the ex-closures as enrichment planting constitutes 24%, 16% and 15% of the total sample standing woody species in the six, fifteen, twenty-five years ex-closures respectively. The rest larger proportion 76%, 84% and 85% of the sample standing woody species in the six, fifteen, twenty-five years ex-closures respectively were not planted which were regenerated indigenous species (Supplementary Table S1).

### Woody species richness and diversity in the soil seed banks

The land-use type had shown a significant difference in the woody species richness and diversity in the soil seed banks (P = 0.0148) and (P = 0.0218) respectively. Thus, the mean ± standard error (SE) woody species richness was (3.7 ± 0.49), (2.9 ± 0.35), (4.3 ± 0.25) and (4.7 ± 0.34) for DOGL, six, fifteen and twenty-five years ex-closures respectively. While, the mean (±SE) woody species diversity was (0.94 ± 0.18), (0.74 ± 0.13), (1.15 ± 0.07) and (1.28 ± 0.07) for DOGL, six, fifteen and twenty five years ex-closures respectively (Table 1). The result through Eq.(1) showed the highest overall Shannon-Wiener diversity index of the soil seed bank was recorded in the DOGL (H’ = 2.03) (Table 1) and the least Shannon-Wiener diversity index was recorded in fifteen years ex-closure.

**Table 1:** Mean (±SE) Shannon-Weiner diversity, richness, and index of woody species in the soil seed banks.

Land-use type	mean (±SE) richness	mean (±SE) diversity	Shannon-Wiener diversity index (H’)
Degraded open grazing land	3.7 ± 0.49	0.94 ± 0.18	2.03
Six years ex-closure	2.9 ± 0.35	0.74 ± 0.13	1.84
Fifteen years ex-closure	4.3 ± 0.25	1.15 ± 0.07	1.78
Twenty-five years ex-closure	4.7 ± 0.34	1.28 ± 0.07	2.01

### The density of seeds in the soil

The overall total seed densities (D) per square metre (m^−2^) were 155 m^−2^, 107 m^−2^, 265 m^−2^ and 275 m^−2^ for DOGL, six, fifteen and twenty-five years ex-closures respectively (Supplementary Table S1). The mean (±SE) seed densities (m^−2^) of soil seeds down to 9 cm of soils were (15.5 ± 3.2), (10.7 ± 3.0), (26.5 ± 12.3) and (27.5 ± 7.7) for DOGL, six, fifteen and twenty five years ex-closures respectively (Table 2). The overall mean densities of the soil seeds did not show significant variation between the land use types (P = 0.1141) (Table 3).

**Table 2:** Mean (±SE) seeds densities m^−2^ recovered from the soil seed banks.

Land-use type	mean (±SE) seeds densities	Layers of soil			
		Litter	0–3 cm	3–6 cm	6–9 cm
Degraded open grazing land	15.5 ± 3.2	1.8 ± 1.2	3.9 ± 1.5	3.9 ± 1.2	5.7 ± 2.0
Six years ex-closure	10.7 ± 3.0	2.1 ± 1.3	2.6 ± 1.2	3.4 ± 1.1	2.1 ± 1.0
Fifteen years ex-closure	26.5 ± 12.3	15.4 ± 7.8	6.2 ± 3.0	3.0 ± 1.7	1.8 ± 1.2
Twenty-five years ex-closure	27.5 ± 7.7	17.3 ± 6.6	3.5 ± 1.6	6.1 ± 2.5	0.4 ± 0.4

**Table 3:** Results of two way ANOVA test to soil seed bank density across soil layers and each land use type.

Source of variations	Df	Sum Sq	Mean Sq	F value	Pr(>F)
Land-use type	3	531	176.8	2.0177	0.1141
Soil Layer	3	1007	335.7	3.830	0.0112*
Soil layer*land-use type	9	1842	204.7	2.336	0.0174*
Residuals	144	12622	87.7		

Significance codes: 0 ‘***’ 0.001 ‘**’ 0.01 ‘*’ 0.05 ‘.’ 0.1 ‘’ 1.

*Carissa edulis* (Forssk.) Vahlexhibited the highest seed density (31 seeds m^−2^) and it accounts for 20% of the total seed density in the DOGL. While the highest density of seeds was scored by *Dodonaea angustifolia*L.f in all ex-closure sites with 27 seeds m^−2^, 133 seeds m^−2^ and 84 seeds m^−2^ in six, fifteen and twenty-five years ex-closures respectively.

### Vertical distribution of seeds in the soil layers

Seeds of woody species in the soil seed banks showed different distributions between the vertical layers of the soil. For instance, the mean (±SE) seeds densities (m^–2^) recovered from the soil seed banks showed the highest (5.7 ± 2.0) in the bottom (6–9 cm) layer and the least (1.8 ± 1.2) in the top (litter) layer of the DOGL (Table 2). In contrary to this, the highest and the least mean (±SE) seed densities (m^–2^) recovered from the soil seed banks of twenty-five years ex-closure was (17.3 ± 6.6) and (0.4 ± 0.4) in the top (litter) layer and in the bottom (6–9 cm) layer respectively. As it is shown in Table 3, the seed densities of the soil seed banks between the land use types did not show a significant difference (P = 0.1141). However, there were significant differences in the seed densities of the soil seed banks between vertical layers of the soil samples (P = 0.0112) and the interaction effect of the land use types and the vertical soil layers (P = 0.0174) (Table 3).

**Figure 1:**
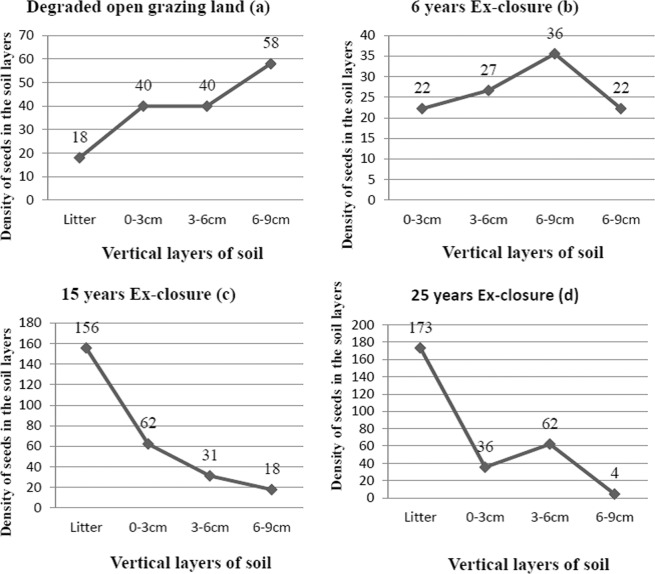
The density of total seeds in the vertical layers of soil in each land-use type.

The pattern of seed density from the top (litter) layer to bottom (6–9 cm) layer of soil samples showed various trends in the land use types (Fig. 1a–d). The highest seed density was scored in the litter layers of ex-closures showing an increasing trend of seed densities in the litter layers of the soil from the youngest ex-closure to the oldest ex-closure (Fig. 1c,d). On contrary to this, as the age of ex-closures increases, seed densities in the mineral (0–9 cm) layers of the soils decreased. However, the litter layer of the soil samples from the DOGL scored the lowest seed density and elevated automatically in deep mineral soil layers resulted in highest seed density in the bottom (6–9 cm) layer of the soil (Fig. 1a).

### Similarities between species compositions of soil seed banks and aboveground woody species

The similarities between the soil seed banks and aboveground vegetation had Jaccard’s Coefficient of similarities (JCS) value of 0.4, 0.2, 0.44 and 0.27 in DOGL, six, fifteen and twenty-five years ex-closures respectively (Table 4). Thus, the lowest JCS was scored in the six years ex-closure and the highest JCS was in twenty-five years.

**Table 4:** Jaccard’s Coefficient of Similarities of woody species composition in the soil seed banks and aboveground woody species.

Land-use type	Jaccard’s similarity index
Degraded open grazing land	0.4
Six years ex-closure	0.2
Fifteen years ex-closure	0.44
Twenty-five years ex-closure	0.27

**Figure 2:**
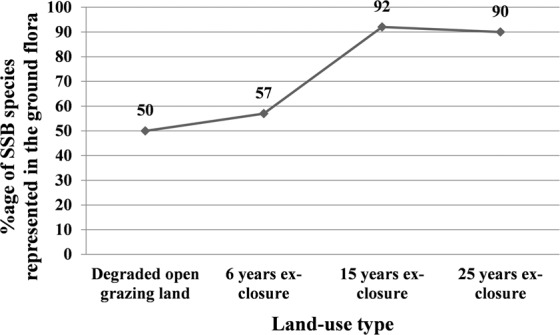
The proportion of soil seed bank species denoted in the above-ground flora of each land-use types.

The number of woody species in the aboveground standing vegetation was much higher than the number of woody species in the soil seed banks of ex-closures. Thus, the proportion of woody species in the soil seed banks which were represented in the aboveground standing vegetation showed substantial variation across the age of ex-closures. For instance, 50%, 57%, 92%, and 90% of all the woody species of the soil seed banks in the DOGL, six, fifteen and twenty-five years ex-closure respectively had represented in the aboveground flora of each site (Fig. 2 and Supplementary Table S2).

## Discussions

The highest overall Shannon-Wiener diversity index of the soil seed bank was recorded in the DOGL (H’ = 2.03) (Table 1) that indicating relatively highest abundance and evenness of woody species was scored in the soil seed banks of DOGL^20^. The overall highest seed density was scored in the fifteen and twenty-five years ex-closures which could be the result of the presence of matured woody species in the old ex-closures that could produce new seeds to the soil. However, the six years ex-closure scored the lowest seed density of all the land use types. This could happen when regeneration of seedlings from the soil seed banks triggered by the establishment of ex-closure is damaged or eaten by livestock which is entered into the ex-closed area illegally. That is, once the seeds in the soil emerged following the conducive environments created due to the establishment of the ex-closures, no more those seeds in the soil seed banks would be available unless other matured trees produce and supply it which is rare in degraded lands.

The number of woody species in the soil seed banks of all ex-closures was lower than the number of their respective aboveground woody species and most of the woody species in the soil seed banks were represented in their respective aboveground woody species. Besides, a larger proportion of the sample standing woody species in the ex-closures were indigenous species regenerated from the soil seed banks following the establishment of the ex-closures. Most of the soil seed bank species were germinated in ex-closures by using the advantages of encouraging environmental conditions like increased soil moisture, organic matter, and other soil nutrients generated following the removal of the external stress by the ex-closure practices^2,18^.

However, the number of woody species in the soil seed banks of DOGL was higher than the number of its respective aboveground woody species and only half of the soil seed bank species in the DOGL were represented in their respective aboveground woody species and the rest kept as viable in the soil seed banks until the surrounding edaphic and climatic conditions improved. Thus, these explicitly verified that soil seed banks well kept up seeds of woody species for the period of unfavourable environmental conditions and played a foremost role in woody species restoration in the degraded lands through ex-closure practices. Information about the past vegetation composition and the structure of a future population can provide by soil seed banks. Similarly, many previous studies agreed that the composition of the seed banks mainly depends on seed production and composition of the current and previous vegetation, as well as on the seed longevity of each species^21,22^.

The variation of representation of most of the woody species of the soil seed banks in the aboveground standing woody species of ex-closures in different ages implies the presence of various longevity potential of seeds of woody species in the soil seed bank environments. Thus, the 10% of the woody species in the soil seed banks which had not denoted in the aboveground flora of twenty-five years ex-closure indicating that some seeds of woody species in the soil seed banks could stay more than twenty-five years as persistent until specific environmental requirements of woody species germinations are changed. The length of time in which seeds can remain viable in the soil can be affected by germination, dormancy, and viability of the seeds, the environmental conditions where seeds available and subsequent changes^23^. Thus, this result indicated that some seeds of woody species in the soil seed banks could stay more than twenty-five years as persistent until specific environmental requirements of the species to germinations are improved.

As the age of ex-closures increases, seed densities in the mineral (0–9 cm) layers of the soils decreased (Fig. 1a–d). Such patterns could happen due to increased woody species coverage in the older ex-closures using the favourable environmental conditions created due to ex-closures resulted in the production and supply of new seeds to the litter layers from matured trees. The increasing trends of seed densities from litter to bottom layers of the soils in the DOGL was seen (Fig. 1a) and could be caused by the absence of enough matured trees in the DOGL that can supply new seeds to the litter layer and/or predation and/or damage of seeds of the available standing woody species by livestock^24,25^.

**Figure 3:**
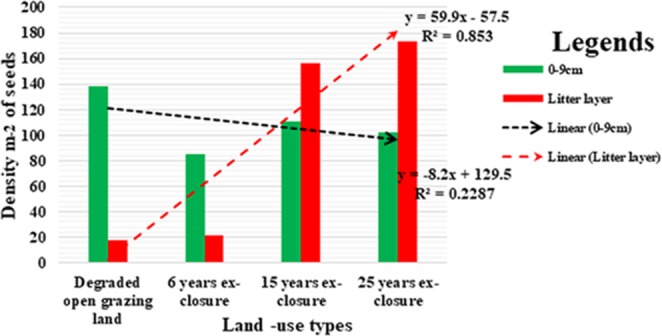
The density (m-2) of seeds in the litter layer and sum total of other layers (0–9 cm).

Even as it is illustrated in Fig. 3, the total seed densities in the entire three lower (0–9 cm) soil layers showed a decreasing trend from DOGL to older ages of ex-closures. While the density of seeds in the litter layers showed an increasing trend from DOGL to older ages of ex-closures. This could happen because seeds in the mineral soil of ex-closures germinated after conducive environments created after the ex-closures establishment and new seeds production from matured trees in the older ex-closures. But, in cases of DOGL, seeds had not germinated but stored in the mineral soil with rare or no production and addition of new seeds on the litter layers. Thus, it clearly showed that persistent seeds of woody species had been preserved in the soil seed banks of degraded lands until edaphic and climatic conditions are changed and would play a vital role in the restoration process.

## Conclusions

Almost all of the soil seed bank species in the older ex-closures were regenerated from the soil seed banks following the establishment of the ex-closures and represented in their respective aboveground standing woody species. However, only half of the soil seed bank species in the degraded open grazing land were denoted in their respective aboveground woody species. The rest half of the soil seed banks kept as viable until the degradation conditions improved. Only 10% of all the woody species in the soil seed banks are represented in the above-ground flora of the twenty-five years ex-closure showing that some seeds of woody species in the soil seed banks could stay more than twenty-five years as persistent until specific environmental requirements of the species to germinations are improved. Therefore, this study undoubtedly verified that soil seed banks kept up seeds of woody species for the period of land degradations until environmental conditions improved and played the foremost role in woody species restoration in the degraded lands through ex-closure practices. Finally, while planning to establish an ex-closure in degraded land, it is also important to examine the level of degradation thereby determining the potential of soil seed banks to regenerate woody species. This could save the costs related to enrichment planting in the degraded lands.

## Materials and Methods

### Study area

**Figure 4:**
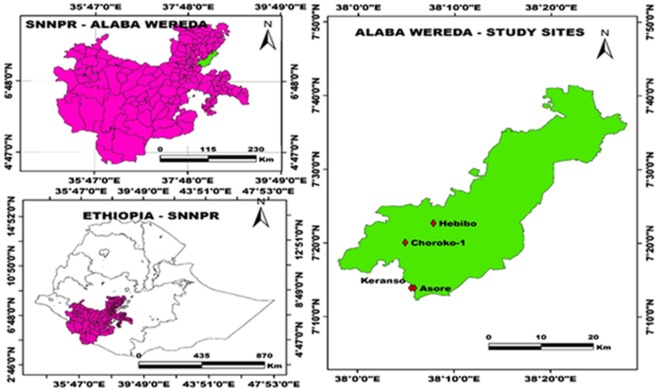
Location map of the study area.

The study area is located in Alaba district in the central Rift Valley of southern Ethiopia (Fig. 4). The area is located 7°18′37″N latitude and 38°5′49″E longitude within the Bilate River Catchment Area. The altitudinal range of the Woreda is 1554–2149 m.a.s. Annual rainfall varies from 857 to 1085 mm, and the annual mean temperature varies from 17° to 20 °C. It is highly likely that much of the Woreda was once covered with closed dry evergreen Afromontane forests^26^. However, deforestation and the expansion of crop production and pasture lands for a long period have reduced to the present forest cover about 7%. Crop cultivation, livestock rearing, and apiculture are the main sources of livelihood. Three ex-closures and one degraded adjacent open grazing land site in similar agro-ecological zones were selected based on their duration of the establishment. These were Choroko-1 (six years), Hebibo (fifteen years), Asore (twenty-five years) ex-closures and degraded open grazing land (Keranso) (Fig. 4).

### Soil seed bank sampling

To collect the soil samples, parallel transect lines were prepared horizontally from one end of the site to the other end in 150 to 300 m width based on the size of the sites. The number of transects per site was based on altitudinal variation and the total area of the site^2,25^. The soil samples were taken from a quadrat of 20 m × 20 m along the transect lines. In the quadrats along the transects, two lines from one corner to the opposite corners were crossed each other and at the four corners and at the intersection point of the two lines at the center of each quadrat 15 cm × 15 cm square was marked (Supplementary Fig. S3) and soil samples removed carefully by using a sharp knife and a spoon and put into plastic bags. The soil samples were carefully separated into four vertical layers (litter layers, 0–3 cm, 3–6 cm and 6–9 cm)^25^. Thus, a total of 160 soil samples (4 land uses x 4 vertically successive layers x 10 soil samples from each site) were collected. The soil samples from similar layers in the quadrats were combined to form a composite soil sample. The litter layer was included with the soil samples as the fourth layer because according to to^23^ all viable seeds and fruits present on or in the soil and associated litter/humus are considered as soil seed banks.

The soil sample collection was completed within three weeks after the starting of it to avoid any temporal bias in seed availability and composition^27^. The samples from each of the soil layers were used to determine variations of seed distribution at each depth of the soil layers^28^ and the samples from each site were used to study the quantity and composition of soil seed banks in the ex-closures of different age and adjacent degraded open grazing land. Soil samples from each layer were coded into plastic bags and transported to Hawassa University, Wondo Genet College of Forestry and Natural Resources for using the greenhouse of the college for incubation and facilitate the germination process of the soil seeds.

### Soil seed identification

Although methods that have been used for determining the number of seeds in the soil seed banks were controversial, the most frequently used techniques for determination of quantity of seeds in the soil were (1) placing soil samples in a suitable places for germination of seeds in the soil (seedling emergence method)^29^ and (2) using physical separation of seeds from the soil particles based on the differences in size and density of the seeds (seed extraction method)^30^. Many researchers prefer the seedling emergence method to seed extraction method assuming that the seedling emergence method is more reliable than seed extraction method^31–33^. Even, the best way to determine the presence and quantity of seeds in the soil is simply observing the seedlings emerging in the sites^11^. Both seedling emergence and sieving methods also used at the same time in different studies of soil seed banks^15,25,34,35^. In this study, both “seed extraction” and “seedling emergence” methods were used for determining the quantity and composition of seeds of the soil seed banks (Supplementary Figs. S1, S2).

### Experimental design

Soil samples were first sieved with a mesh size of 2 mm and then using a mesh size of 0.5 mm to recover seeds of various woody species^36,37^. All the seeds recovered from the sieving process were subjected to viability tests through dissecting of the seeds in which seeds with white and firm content were considered viable^15,25,38^. Then all viable seeds in each sample were counted^25^. The recovered seeds were collected into paper bags and taken back to the study area for species identification by using seeds of standing vegetation as a local reference.

Soil samples remained after sieving were taken to germination trials in a greenhouse (Fig. S1) to control and detect contamination of external seed rain. The soil samples were spread in plastic trays that pierced at the bottom to facilitate proper drainage of water for germination of the seeds Daily temperature for the greenhouse ranged from 19 to 32 °C. The seedling trays were kept continuously moist by daily watering^27^. The soil samples were stirred once in two weeks stimulating them to germinate because mixing is known to cause more seeds to germinate^39^. The emerging seedlings which were readily identifiable were counted, recorded and discarded^25^. Seedlings that were difficult to identify were transplanted for further growth and identification. Seedling identification was terminated after 6 months.

### Aboveground woody species sample data collection

The aboveground woody species (trees and shrubs) data collected from the three ex-closures under different ages and adjacent degraded open grazing land. A total of 120 quadrats 30 from each laid along transect lines having a quadrate size of 20 m × 20 m (400 m^2^). The altitude of each quadrate was recorded by using a global positioning system (GPS) devices 3 m level of accuracy. The total numbers of each woody species in each quadrat were recorded. Woody species encountered were identified by their vernacular and scientific names based on own experience supported by plant knowledge of local elders^40,41^ and the Flora of Ethiopia and Eritrea^42–44^. For species that were difficult to identify in the field, herbarium specimens were collected, pressed, dried and transported to the National Herbarium in Addis Ababa University, for proper identification.

### Data analysis

The species richness (S), diversity and density of seeds in the soil were determined by combining the data obtained by “seed extraction” and “seedling emergence” methods. The density of seeds was derived from the total number of seeds recovered from the soil samples. On the other hand, to analyze the depth distribution of seeds in each site, the number of seeds recovered in similar layers were combined and converted to provide the density of seeds m^−2^ at that particular soil depth^45^.

The species diversity and evenness of seeds recovered from the soil seed banks from the four study sites were calculated using the Shannon-Wiener index^46^:

1 $${\rm{Shannon}}\,{\rm{diversity}}\,{\rm{index}},\,{\rm{H}}^{\prime} =-\,\mathop{\sum }\limits_{i=1}^{s}\,p{\rm{i}}\,\mathrm{ln}\,p{\rm{i}}$$

Where: H′ = species diversity index; ln = natural logarithm and Pi = n/N is the proportion of individuals found in the i^th^ species (ranges 0 to 1); n = number of individuals of a given species; and N = total number of individuals found in that particular site.

The significance of mean species richness and diversity between the land use types were compared using one-way ANOVA. While the mean differences of seed densities concerning the land use types and vertical layers of the soil were compared using two-way ANOVA. Significance was determined at an alpha level of 0.05. All the data obtained were analysed by statistical software R version 3.4.1. The similarity of the species composition between soil seed banks and that of the standing vegetation was calculated with Jaccard’s coefficient of similarity (JCS)^47^.

## Supplementary information

Supplementary Information.

## Data Availability

The datasets generated during and/or analysed during the current study are available from the corresponding author on reasonable request.
