# Supplementary material for: Study of Soil Seed Banks in Ex-closures for Restoration of Degraded Lands in the Central Rift Valley of Ethiopia
Source: Sci Rep. 2020 Jan 22;10:956. doi: 10.1038/s41598-020-57651-1 (PMC6976692; doi:10.1038/s41598-020-57651-1)
Supplement: Supplementary file 1 — Supplementary Information. [file 41598_2020_57651_MOESM1_ESM.pdf]

Study of Soil Seed Banks in Ex-closures for Restoration of Degraded Lands in the Central Rift Valley of Ethiopia

Shemsedin Ahmed Mohammed*[1, 2] and Mekuria Argaw Denboba[2]

[1]Department of Environmental Science, College of Natural and Computational Science, Wolaita Sodo University, Ethiopia

[2]Center for Environmental Science, College of Natural and Computational Sciences, Addis Ababa University, Ethiopia

Correspondence to Shemsedin Ahmed at Center for Environmental Science, College of Natural and Computational Sciences, Addis Ababa University, Ethiopia. E-mail: shemsu1985@gmail.com

Supplementary Table 1. Total woody species identified and their density (D) (m^{-2}) in the soil seed banks

**Name of the Species**	**Family**	**Life form**	**Land-use type**			
			**Degraded open grazing land**	**Six years ex-closure**	**Fifteen years ex-closure**	**Twenty-five years ex-closure**
			**D (m^{-2})**	**D (m^{-2})**	**D (m^{-2})**	**D (m^{-2})**
Acacia torotolis (Forssk.) Hayne	Mimosaceae	Tree	18	18	9	13
Acacia persiciflora Pax	Fabaceae	Tree	22	18	0	0
Acacia seyal Del.	Fabaceae	Tree	18	0	9	0
Acokanthera schimperi (A.DC.) Schweinf.	Apocynaceae	Shrub	0	13	13	18
Balanites aegyptiaca (L.) Del.	Balanitaceae	Tree	0	0	13	22
Calpurnia aurea (Ait.) Benth	Fabaceae	Shrub	9	9	4	22
Carissa edulis (Forssk.) vahl	Apocynaceae	Shrub	31	4	9	0
Cordia Africana Lam.	Boraginaceae	Tree	0	0	0	13
Croton macrostachyus Hochst.ex Del.	Euphorbiaceae	Tree	0	0	4	0
Dichrostachys cinerea (L.) Wight and Arm	Fabaceae	Tree	13	0	9	9
Dodonaea angustifolia L.f	Sapindaceae	Shrub	22	27	133	84
Eucalyptus camaldulensis Dehnh.	Myrtaceae	Tree	0	0	40	58
Ficus sur Forssk.	Moraceae	Tree	0	0	9	0
Jacaranda mimosifolia Guenter.	Bignoniaceae	Tree	22	0	0	0
Olea europaea Mill.	Oleaceae	Tree	0	18	13	27
Podocarpus falcatus (Thumb.) R.B.ex.Mirb.	Podocarpaceae	Tree	0	0	0	9
Total number of species			**8**	**7**	**12**	**10**
Over all total density			**155**	**107**	**265**	**275**

Supplementary Table 2. List of woody species in standing vegetation and soil seed banks

**Degraded open grazing land**	
**Species in the above-ground vegetation**	**Species in the Soil Seed bank**
Acacia seyal Del.	*Acacia torotolis* (Forssk.) Hayne
Acacia tortilis (Forssk.) Hayne	*Calpurnia aurea* (Ait.) Benth
Calpurnia aurea (Ait.) Benth	*Carissa edulis* (Forssk.) vahl
Carissa edulis (Forssk.) vahl	*Dichrostachys cinerea* (L.) Wight & Arn.
Croton macrostachyus Hochst.ex Del.	*Dodonaea angustifolia* L.f
Ficus sur Forssk.	*Jacaranda mimosifolia* Guenter.
	Acacia persiciflora Pax
	Acacia seyal Del.
**Six years ex-closure**	
**Species in the above-ground vegetation**	**Species in the Soil Seed bank**
Acacia polycantha (A. Rich.) Brenan	*Acacia torotolis* (Forssk.) Hayne
Acacia saligna (Labill.) Wendl.f.	*Acacia persiciflora* Pax
Acacia seyal Del.	*Acokanthera schimperi* (A.DC.) Schweinf.
Acacia sieberiana DC.	*Calpurnia aurea (Ait.)* Benth
Acacia tortilis (Forssk.) Hayne	*Carissa edulis (Forssk.)* vahl
Balanites aegyptiaca (L.) Del.	*Dodonaea angustifolia* L.f
Calpurnia aurea (Ait.) Benth	*Olea europaea* Mill.
Carissa edulis (Forssk.) vahl	
Cordia Africana Lam.	
Croton macrostachyus Hochst.ex Del.	
Dichrostachys cinerea(L.) Wight & Arn.	
Dodonaea angustifolia L.f	
Eucalyptus camaldulensis Dehnh.	
Grevillea robusta A.Cunn. Ex R.Br.	
Jacaranda mimosifolia Guenter.	
Rhus natalensis Bernh.ex Krauss	
Vachellia seyalv (Delile) P.Hurter	
**Fifteen years ex-closure**	
**Species in the above-ground vegetation**	**Species in the Soil Seed bank**
Acacia abyssinica Hochst. Ex Benth	*Acacia torotolis* (Forssk.) Hayne
Acacia persiciflora Pax	*Acacia seyal* Del.
Acacia saligna (Labill.) Wendl.f.	*Acokanthera schimperi* (A.DC.) Schweinf.
Acacia seyal Del.	*Balanites aegyptiaca*
Acacia sieberiana DC.	*Calpurnia aurea* (Ait.) Benth
Acacia tortilis (Forssk.) Hayne	*Carissa edulis* (Forssk.) vahl

Acokanthera schimperi (A.DC.) Schweinf.	*Croton macrostachyus* Hochst.ex Del.
Balanites aegyptiaca (L.) Del.	*Dichrostachys cinerea* (L.) Wight &Arn.
Calpurnia aurea (Ait.) Benth	*Dodonaea angustifolia* L.f
Carissa edulis (Forssk.) vahl	*Eucalyptus camaldulensis* Dehnh.
Casuarina cunninghamiana Miq.	*Ficus sur* Forssk.
Celtis Africana Burm. F.	*Olea europaea* Mill.
Croton macrostachyus Hochst.ex Del.	
Dichrostachys cinerea (L.) Wight &Arn.	
Dodonaea angustifolia L.f	
Eucalyptus camaldulensis Dehnh.	
Ficus sycomorus L.	
Grevillea robusta A.Cunn. Ex R.Br.	
Grewia ferruginea Hochst. Ex A. Rich	
Leucaena leucocephala (Lam.) De Wit.	
Maerua angolensis DC	
Olea europaea Mill.	
Rhus natalensis Bernh.ex Krauss	
Vachellia seyal (Delile) P.Hurter	
**Twenty-five years ex-closure**	
**Species in the above-ground vegetation**	**Species in the Soil Seed bank**
Acacia albida Del.	*Acacia torotolis* (Forssk.) Hayne
Acacia saligna (Labill.) Wendl.f.	*Acokanthera schimperi* (A.DC.) Schweinf.
Acacia senegal (L.) Willd.	*Balanites aegyptiaca* (L.) Del.
Acacia seyal Del.	*Calpurnia aurea* (Ait.) Benth
Acacia sieberiana DC.	*Cordia africana* Lam.
Acacia tortilis (Forssk.) Hayne	*Dichrostachys cinerea* (L.) Wight &Arn.
Acokanthera schimperi (A.DC.) Schweinf.	*Dodonaea angustifolia* L.f
Balanites aegyptiaca (L.) Del.	*Eucalyptus camaldulensis* Dehnh.
Calpurnia aurea (Ait.) Benth	*Olea europaea* Mill.
Carissa edulis (Forssk.) vahl	*Podocarpus falcatus* (Thumb.) R.B.ex.Mirb.
Casuarina cunninghamiana Miq.	
Celtis africana Burm. F.	
Clerodendrum myricoides (Hochst.) Vatke	
Cordia africana Lam.	
Croton macrostachyus Hochst. ex Del.	
Dichrostachys cinerea (L.) Wight & Arn.	
Diospyros abyssinica (Hiern) F. White	
Dodonaea angustifolia L.f	
Ehretia cymosa Thonn.	
Erythrococca abyssinica Pax	

Eucalyptus camaldulensis Dehnh.	
Flacourtia indica (Burm. f.) Merr.	
Grevillea robusta A.Cunn. Ex R.Br.	
Grewiaferruginea Hochst. Ex A. Rich	
Jacaranda mimosifolia Guenter.	
Maerua angolensis DC	
Maytenus arbutifolia (A. Rich.) Wilczek	
Maytenus senegalensis (Lam.) Exell	
Olea europaea Mill.	
Osyris quadripartite Decn.	
Rhus natalensis Bernh.ex Krauss	
Rhus glutinosa A.Rich.	
Vachellia seyal (Delile) P.Hurter	

D = Density of seeds per m^2

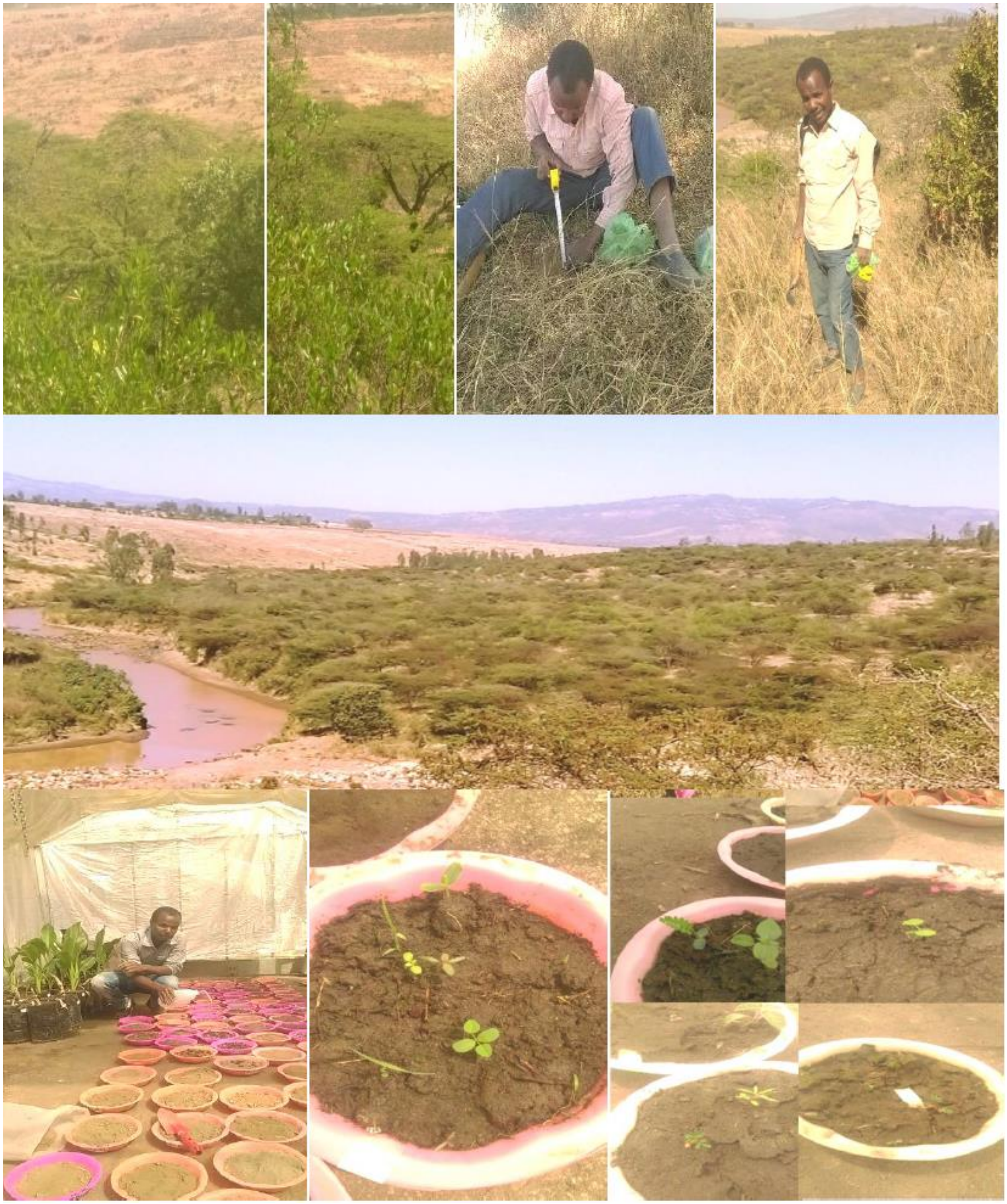

Supplementary Figure 1. Soil seed bank sampling and woody species identification through “seedling emergence” method in a greenhouse

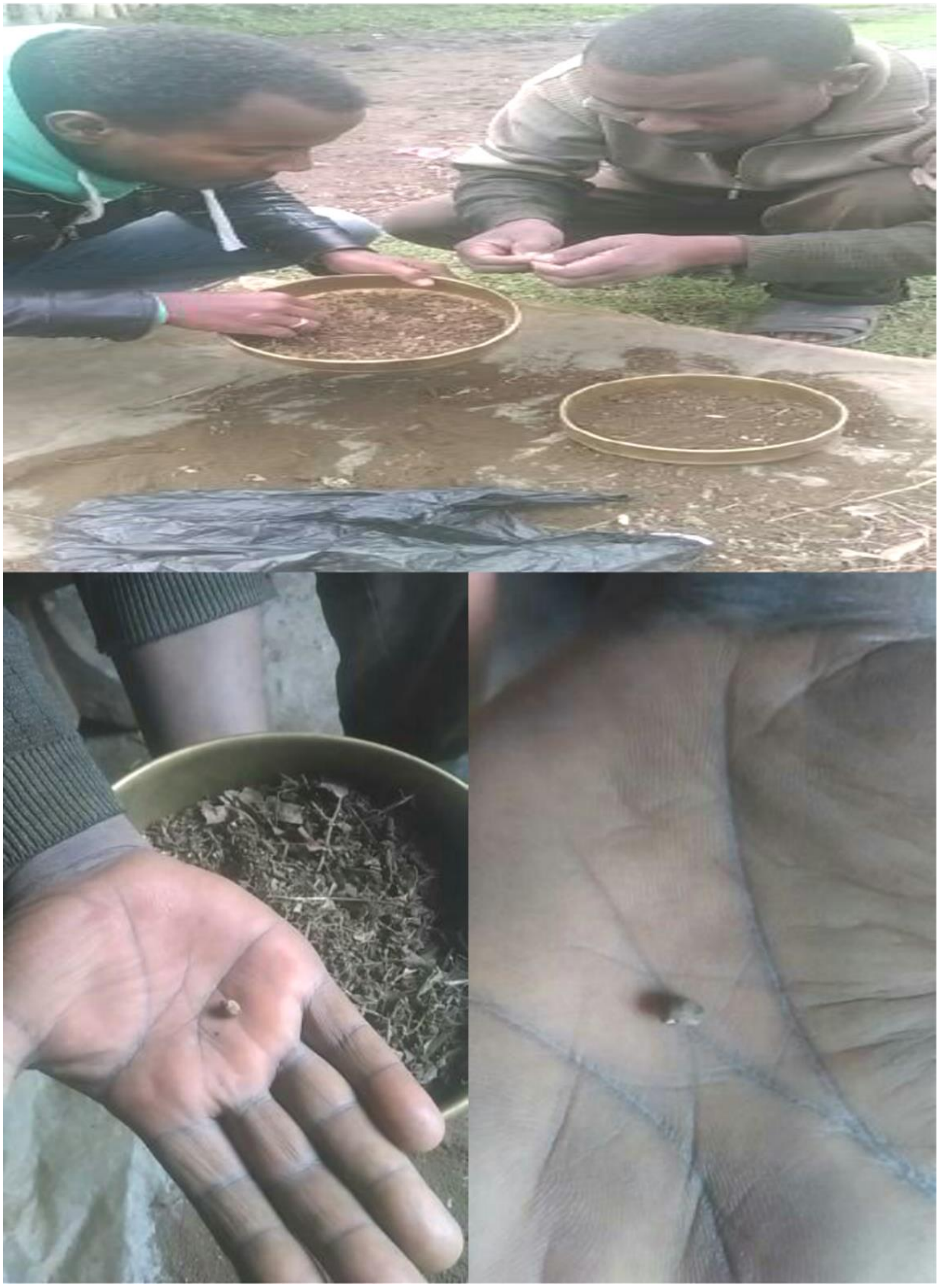

Supplementary Figure 2. Woody species identification through “seed extraction” method

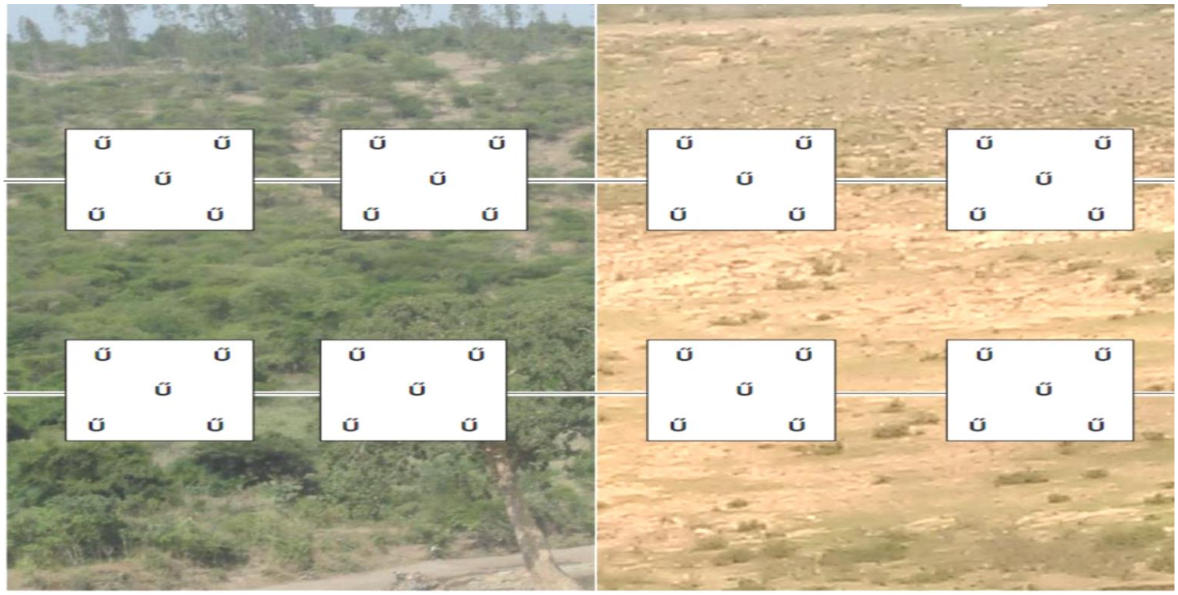

Supplementary Figure 3. Diagrammatic sketch of soil sampling design
